# Increased expression levels of Syntaxin 1A and Synaptobrevin 2/Vesicle-Associated Membrane Protein-2 are associated with the progression of bladder cancer

**DOI:** 10.1590/1678-4685-GMB-2017-0339

**Published:** 2019-01-21

**Authors:** Sadaf Azad Raja, Seher Abbas, Syed Tahir Abbas Shah, Aamira Tariq, Nazia Bibi, Arzu Yousuf, Athar Khawaja, Muhammad Nawaz, Arshad Mehmood, Muhammad Jadoon Khan, Alamdar Hussain

**Affiliations:** 1 COMSATS Institute of Information Technology COMSATS Institute of Information Technology Department of Biosciences Islamabad Pakistan Department of Biosciences, COMSATS Institute of Information Technology Islamabad, Pakistan; 2 Shifa International Hospital Shifa International Hospital Department of Urology and Kidney Transplant Islamabad Pakistan Department of Urology and Kidney Transplant, Shifa International Hospital, Islamabad, Pakistan; 3 Armed Forces Institute of Urology Armed Forces Institute of Urology Rawalpindi Pakistan Armed Forces Institute of Urology (AFIU), Rawalpindi, Pakistan

**Keywords:** SNARE, bladder cancer, vesicle fusion, gene expression

## Abstract

Gene expression is tightly regulated in time and space through a multitude of factors consisting of signaling molecules. Soluble N-ethylmaleimide-sensitive-factor attachment protein receptors (SNARE) are membrane proteins responsible for the intercellular trafficking of signals through endocytosis and exocytosis of vesicles. Altered expression of SNARE proteins in cellular communication is the major hallmark of cancer phenotypes as indicated in recent studies. SNAREs play an important role in maintaining cell growth and epithelial membrane permeability of the bladder and are not only involved in cancer progression but also metastatic cell invasion through SNARE-mediated trafficking. Synaptobrevin2/Vesicle associated membrane protein-2 (v-SNARE) and Syntaxin (t-SNARE) form a vesicular docking complex during endocytosis. Some earlier studies have shown a critical role of SNARE in colon, lungs, and breast cancer progression and metastasis. In this study, we analyzed the relative expression of the *STX1A* and *VAMP2* (*SYB2*) for their possible association in the progression and metastasis of bladder cancer. The profiling of the genes showed a significant increase in *STX1A* and *VAMP2* expression (*p* < 0.001) in high-grade tumor cells compared to normal and low-grade tumors. These findings suggest that elevated expression of *STX1A* and *VAMP2* might have caused the abnormal progression and invasion of cancer cells leading to the transformation of cells into high-grade tumor in bladder cancer.

## Introduction

Transitional cell carcinoma is the common form of histologic bladder cancer (90% cases) and has significant mortality rate (77.89% 5-year relative survival) (American Cancer Society, 2017). High grade tumors have high probability of recurrence with high percentage of progression while low grade tumors have low frequency of recurrence and are less progressive (Miyamoto *et al.*, 2010). According to WHO in 2004 (WHO/International Society of Urological Pathology (ISUP) classification), the classification of bladder cancer is useful in differentiating carcinomas for prognostic evaluation (Pan *et al.*, 2010). Low grade papillary urothelial carcinomas (LPUCs) and high grade papillary urothelial carcinomas (HPUCs) have distinct cancer progression categories and recurrence, and therefore, WHO recently recommended the staging of bladder cancer into only two categories: low grade and high grade (Miyamoto *et al.*, 2010; Pan *et al.*, 2010).

In almost all of the cancers, signal transduction dysregulation has a key role in triggering the cell for survival in malignant conditions (Bartsch *et al.*, 2010). The tumor microenvironment plays a crucial role in maintaining the tumor growth, progression, and metastasis via exploiting growth factors, enzymes, and other signaling molecules that are preferably transported via exosomes (Kang *et al.*, 2017). Proteome analysis of extracellular vesicles (EVs) secreted by the epithelial membrane in muscle invasive bladder cancer (MIBC) showed that these vesicles contain a number of proteins and signaling molecules that are transported to extracellular matrix (ECM) (Silvers *et al.*, 2017). The vesicle trafficking is basically controlled by regulatory receptor proteins present on the membrane of the targeting cell, functioning with the aid of gated channels (Palfreyman and Jorgensen, 2008). Membrane trafficking in the eukaryotic cell is mediated by a SNARE complex [soluble (N-ethylmaleimide-sensitive factor) attachment protein receptors] (Shukla *et al.*, 2000).

The SNARE complex is divided into two groups according to function and location. t-SNAREs are target membrane receptors while v-SNAREs are vesicular membrane proteins (Polchi *et al.*, 2013). Syntaxin-1 (*STX1*) and *SNAP25* are t-SNAREs, resident on the target cell membrane and participate in vesicle fusion whereas *VAMPs* (vesicle associated membrane proteins) are v-SNAREs, anchored to the membrane vesicles excreted or exocytosed by the cell (Haberman *et al.*, 2012; Meng and Wang 2015). The vesicular fusion is accomplished by core SNARE complex comprising such as syntaxin (*STX*), SNAP25, and synaptobrevin2 (*VAMP2*) that mediate final vesicle fusion (Fang and Lindau, 2014).

SNARE proteins are known to actively derive vesicular trafficking between the cells to maintain the cell integrity via cell growth, migration, and wound healing in a regulative manner (Tian *et al.*, 2014). Delivery of extracellular matrix (ECM) and integrins through vesicular transport is the fundamental function of SNARE proteins during cell proliferation and motility. Though SNARE-mediated exosome transport of integrins is critical for cancer development, epidermal growth factors at the cell surface have a major role in ECM regulation, cell survival, and progression (Enrich *et al.*, 2015). SNARE proteins regulate matrix degradation and allow cell migration/invasion (Williams *et al.*, 2014). Functional silencing of SNARE proteins decreases the ability of breast cancer cells to invade and migrate (Steffen *et al.*, 2008). Inhibition of SNARE proteins impairs the development of invadopodium, disrupts cell invasion, and inhibits migration in tumors (Williams and Coppolino, 2014). Altered expression of the SNARE complex has been found critical for various cancers as they are the core signaling proteins involved in vesicular fusion and known to be good targets for cancer therapy (Meng and Wang, 2015). 

STX1A and VAMP2 are known neuronal SNAREs that mediate synaptic vesicular fusion (Ramakrishnan *et al.*, 2012). *STX1A* overexpression has also been observed in primary brain tumor and colorectal, lung, and breast cancers (Grabowski *et al.*, 2002; Arsenault *et al.*, 2013; Fernández-Nogueira *et al.*, 2015). Blocking of *STX1A* inhibits tumor growth in glioblastoma (Ulloa *et al.*, 2015). Little is known about the expression pattern of *VAMP2* in breast and lung cancers and also in bladder cancer. However, loss of *VAMP2* in neuronal tissue leads to endolysosomal degradation. *VAMP2* relies on its sorting behavior for vesicle exocytosis and fusion with target sites. Decreased expression of *VAMP2* causes abnormalities in the degradation pattern of useless proteins (Haberman *et al.*, 2012). Heterogenic expression of *VAMP2* and other SNARE proteins was found in undifferentiated colorectal carcinomas (Grabowski *et al.*, 2002). Importantly, *VAMP2* is known to be involved in the integrin trafficking and critical for cancer cell adhesion, survival, and migration (Hasan and Hu, 2010). SNAREs are thus basic complexes in exosome-mediated cellular communication that regulate the cell cycle and progression (Polchi *et al.*, 2013). According to recent studies on the role of neuronal SNARE complex, specifically *STX1A* and *VAMP2*, in cancers other than brain tumors as regulators of important cellular mechanism of vesicular exocytosis, the heterogenic expression of these genes may cause cellular abnormalities leading to cancer development. Therefore, we aimed to determine the difference in expression of *STX1A* and *VAMP2* in relation to tumor grades and pathological stages in bladder cancer.

## Material and Methods

### Tumor sampling

Tumor and normal tissue samples were collected from post-surgical bladder cancer specimens. The study was approved by the ethical review board (ERB) of COMSATS Institute of Information Technology (No. CIIT/Bio/ERB/18/76). The data were obtained with the written consent of the patients involved in the study. Disease histories were confirmed by the Department of Pathology, Pakistan Institute of Medical Science (PIMS). The total number of samples was 55, out of which 26 were paired. Surrounding normal tissue samples were used as controls. The histopathology reports of the patients were obtained from the Department of Urology PIMS and Shifa International Hospital, Islamabad, Pakistan, for categorizing the tumor samples according to their grades and cancer stage.

### Quantitative PCR

**Figure 1:**
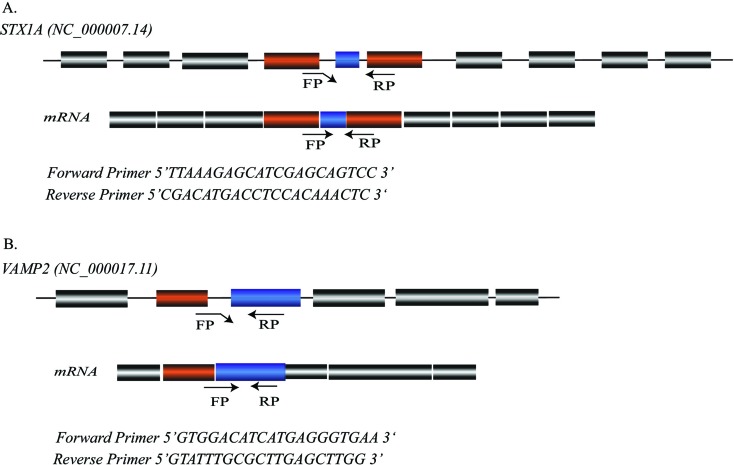
Primer location and specificity for qPCR analysis. (A) Primer pair sequence for *STX1A* (NC_000007.14) (FP 5’ TTAAAGAGCATCGAGCAGTCC 3’ and RP 5’ CGACATGACCTCCACAAACTC 3’), Amplicon size is 120 bp, TM=62 °C, location at GRCh38.p12; (Chr7: 73704218-73704237) (Chr7: 73704423-73704410). (B) *VAMP2 (*NC_000017.11*)* primer pair sequence; forward primer (5’ GTGGACATCATGAGGGTGAA 3’), reverse primer (5’GTATTTGCGCTTGAGCTTGG 3’). Amplicon size is 138 bp, TM=55 °C, location GRCh38.p12; (Chr17: 8161626-8161645), (Chr17: 8161763-8161744).

RNAlater^®^ (Ambion, Thermo Fisher Scientific Inc. Waltham, MA USA) was used to preserve samples. RNA was isolated using Trizol (Thermo Fisher Scientific) reagent according to manufacturer’s protocol (Rio *et al.*, 2010). Quantified RNA (1-2 μg) was used for cDNA synthesis (Thermo Fisher Scientific). Primers for the target genes *STX1A*, *VAMP2* were designed using Primer Quest tool (Integrated DNA Technologies) and further edited to acquire specifications. *TUB3* was used as the endogenous housekeeping gene. The primers designed were specific to *STX1A-001* and *VAMP2-001*. Primer sequences and specification are given in Figure 1. UCSC *in silico* PCR was done using the set of primers to assure amplification. Quantitatve analysis of the expression level of the target genes was done by quantitative real-time PCR in a StepOnePlus Real-Time PCR system (Applied Biosystems). The experiment was run for three biological replicates with negative controls. A melting curve analysis for each sample was performed to check for non-targeted fragment amplification. The volume per reaction was adjusted to 25 μL using Maxima Syber Green/ROX qPCR Master Mix (Thermo Scientific), and cDNA was used at a concentration of 2 μg/μL for each sample. The relative fold-increase in the expression of the *STX1A* and *VAMP2* genes was analyzed using the 2^-DDCT^ method (Livak and Schmittgen, 2001). The data were normalized with the internal control *TUB3* and the average fold-increase was determined by calculating the relative expressions of each tumor sample.

### Statistical analysis

Statistical analyses were performed with OriginPro 2017 (OriginLab, Northampton, MA). For expression data, the C_t_ values of the target genes *VAMP2 and STX1A* were normalized with the control gene (*TUB3*) C_t_. Normality of the data was assessed by the Shapiro-Wilk test. The correlation among different factors was assessed by the Spearman Correlation Coefficient test. Depending on the experiment, the statistical significance was determined using the Wilcoxon, Mann-Whitney, or Kruskal-Wallis ANOVA test, and specific comparisons were made by the Tukey’s Honestly Significant Difference (HSD) test. Fisher’s exact two-tailed test was performed to calculate patient data contingency, with *p* < 0.05 considered as significant.

## Results

### Tumor grade and stage

Out of 55 bladder tumor samples, 31 were high grade and 24 were low grade according to the WHO/ISUP classification system (Miyamoto *et al.*, 2010). The histopathology reports of the patients were obtained from the hospitals (Pakistan Institute of Medical Sciences and Shifa International Hospital, Islamabad Pakistan) and, according to the histopathology examination; the tumors that had recurrent behavior were categorized as high grade tumors. The samples were confirmed as transitional cell carcinomas by the Department of Histopathology (PIMS). There were 10 high grade tumors that had metastasized to the pelvic wall and prostate gland. Among the low grade tumors there were seven tumors that had spread only to the sub-epithelial connective tissue of the bladder. The staging of tumors was based on the TNM staging system (Table 1). The significance and distribution of tumor grade among age intervals was calculated using the Chi-square test (Table 2).

**Table 1:** Histopathology of tumor samples according to TNM staging system.

			Histopathological staging					
			I		II		III	
Grades	Gender	Total NO.	Ta N0 M0	Tis N0 M0	T1 N1 M0	T2 N2 M0	T2a N1 M0	T4a N3 M1
High grade	Female	11	0	1	2	3	3	2
Male	20	0	1	1	4	5	9	
Low grade	Female	8	2	4	2	0	0	0
Male	16	5	3	5	3	0	0	

Ta, Noninvasive papillary carcinoma; Tis, non-muscle-invasive bladder cancer; T4a, Tumor spread to the uterus or prostate; N0, Cancer not spread to regional lymph node/s; N1, Cancer spread to single regional lymph node in pelvis; M0, Non-metastasized; M1, Metastasized to pelvic organs.

**Table 2:** Significance and distribution of tumor grade in young age and older age group.

Patient data	Age interval		*p-v*alue
	35-40 years	> 40 years	
Gender			
Male	24	12	0.5655
Female	11	8	0.5655
High grade tumor			
Muscle invasive	17	9	0.1313
Non-muscle invasive	4	1	0.1313
Low grade tumor			
Muscle invasive	1	2	0.1937
Non-muscle invasive	16	5	0.1937

### Expression of *STX1A* and *VAMP2*

**Figure 2:**
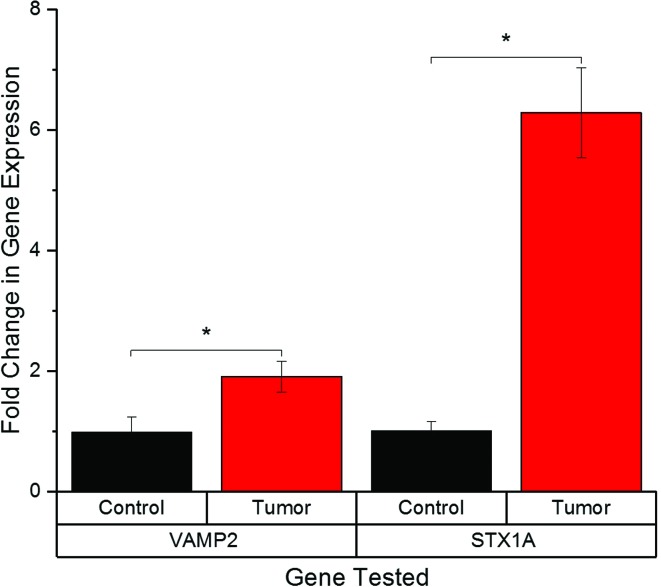
Synaptobrevin2 and Syntaxin1A expression in tumor and adjacent normal bladder tissues. Bar graph of normalized (mean ± SE) gene expression of Synaptobrevin2 and Syntaxin1A, showing a significant increase in tumor tissue compared to adjacent normal tissue.

The relative fold-change in the expression of the *STX1A* was analyzed using the 2^-DDCT^ method (Livak and Schmittgen, 2001). The data were normalized with the internal control gene tubulin (*TUB3),* and the average fold-change was determined by calculating the relative expressions of each tumor sample (Table S1). The relative RNA level of *STX1A* showed a five-fold increase in tumors compared to their controls (*p* < 0.005). Similarly, the expression of *VAMP2* was 2.9-fold higher in tumor samples compared to their controls (*p* < 0.001) (Figure 2).

### Expression of *STX1A* and *VAMP2* relative to tumor grades

**Figure 3:**
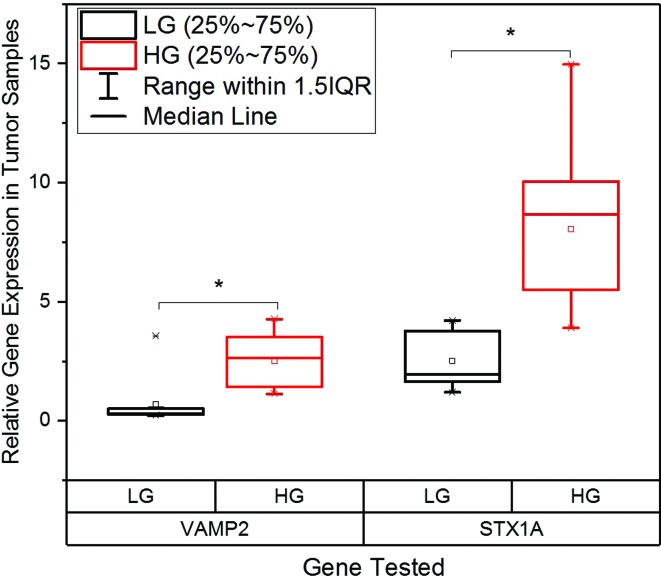
Expression of Synaptobrevin2 and Syntaxin1A in low and high grade tumor tissues. Boxplots of normalized (relative) gene expression of Synaptobrevin2 and Syntaxin1A showing significantly higher expression in high grade tumors compared to low grade tumors.

Expression levels of the genes were correlated to tumor grades (Table S2). According to the pathological grading of bladder cancer, the expression of *STX1A* was highly increased in the high grade invasive tumors with distant metastasis. The expression of *STX1A* was eight-fold higher in high grade tumors and 2.5-fold higher in low grade tumors. Therefore, a significant difference of *STX1A* expression was observed between the high grade and low grade tumors (*p* < 0.001). *VAMP2* expression was also significantly increased in high grade tumors compared to the low grade tumors (*p* < 0.001). Low grade tumors had a lesser fold-increase in the expression of *STX1A* and *VAMP2,* whereas high grade tumors showed relatively higher fold-increases in the expression of both genes (Figure 3). These results suggest that the genes had higher expression in higher grades tumors. The expression of both genes in the controls was normal, indicating that there wass no genetic aberration that might have caused the tumors to progress to high grade.

### Expression of *STX1A* and *VAMP2* relative to tumor stages

**Figure 4:**
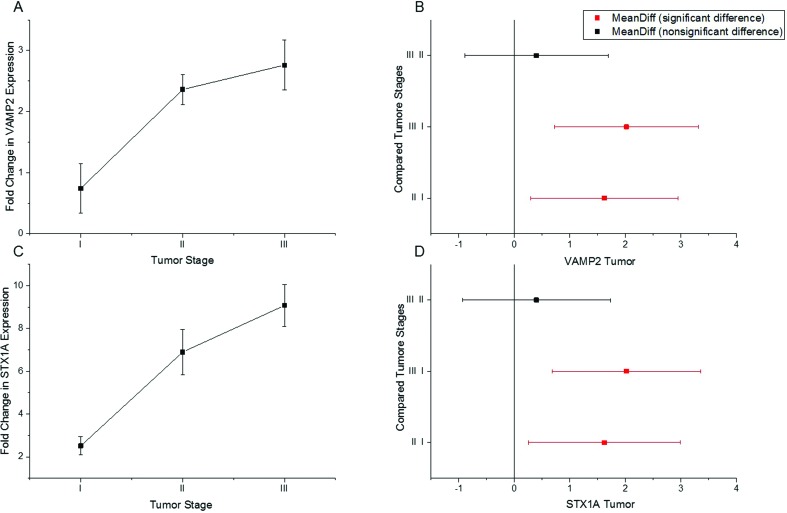
Tumor stage-dependent gene expression. (A and C) Shown is the increase in expression of Synaptobrein2 and Syntaxin1A in a tumor stage-dependent manner (lowest in I and highest in III). (B) A significant difference in the expression of Synaptobrevin2 is seen between stages I and II, and stages I and III, whereas no difference is observed between stage II and III. (D) A significant difference is seen in the expression of Syntaxin1A between stages I and II, and stages I and III, whereas no difference is observed between stages II and III.

The increased expression of both genes was positively correlated to tumor stage (Table S3). Our results showed that the expression of *STX1A* and *VAMP2* increased progressively according to the stage of the tumor (Figure 4A, C). Stage II tumors are invasive and show invasion in the bladder muscles, while stage III tumors are highly invasive and tend to spread in adjacent organs. In another study, no change in expression levels was found for both genes between stage II and III (Lopez-Beltran 2008). In our study we observed a significant difference in the expression levels of the two genes between stage I and III, suggesting that the expression of *STX1A* and *VAMP2* increases in a tumor in a stage-dependent manner (Figure 4B and 4D).

### Expression correlation between *STX1A* and *VAMP2*

**Figure 5:**
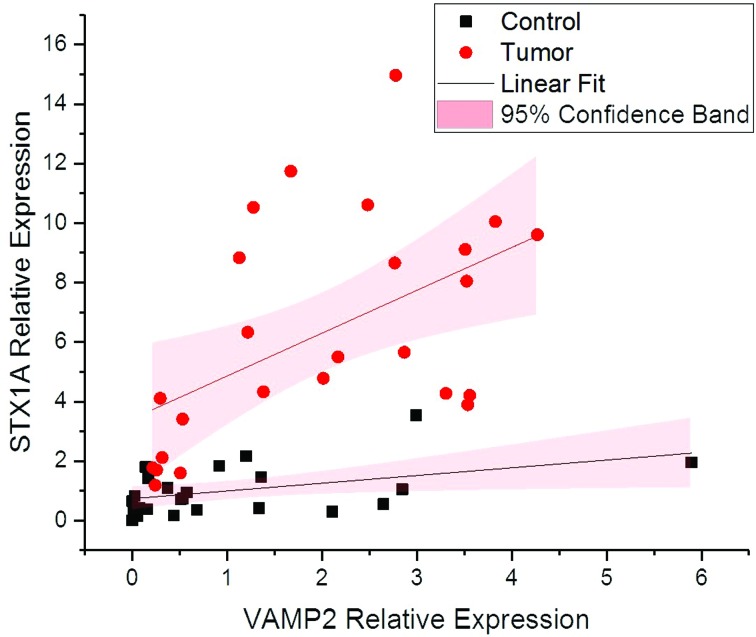
Correlation analysis between expression levels of the Synaptobrevin2 and Syntaxin1A in tumor and adjacent normal bladder tissue. The graph shows a significant positive correlation between expression of Synaptobrevin2 and Syntaxin1A in both tumor and adjacent normal bladder tissues.

Both genes are a crucial part of SNAREs, as vesicle fusion only takes place followed by their interaction forming a vesicle fusion complex. It was previously reported that cancer progression might have a role in the increased expression of the two genes that mediate cell communication through vesicle fusion (Meng and Wang, 2015). These results suggest that the enhanced expression of *STX1A* and *VAMP2* might have role in triggering tumor progression in high grade stage III tumors. In our correlation analysis results, the increase in the expression of both genes was linear (Table S4) according to tumor grade and stage, which determines the strongly positive linear correlation between the two genes (Figure 5)

## Discussion

Epithelial cells along the inner surface of organs form a primary barrier where absorption, secretion, fusion of extracellular vesicles, and exocytosis of exosomes takes place. These cells have a regulatory vesicular communication that is accomplished by a complex of SNARE proteins. SNARE proteins are responsible for maintaining the permeability of the bladder epithelium (Born *et al.*, 2003). The inner luminal membrane exposed to urine releases small vesicles, detaching the membrane that has been subjected to prolonged exposure to urine. Continuous extruding of the apical membrane is regulated by endocytosis of the vesicles (Hurst *et al.*, 2015).

Vesicle trafficking is regulated by SNARE proteins. The SNARE proteins VAMPs (VAMP2) and SXT1A were found to be present in the epithelial fraction of the bladder (Born *et al.*, 2003). STX1A*,* being t-SNARE, accomplishes vesicle fusion while VAMP2, a v-SNARE, plays a key role in Ca^+2^-dependent-exocytosis of the vesicles (Chang and Jackson, 2015). STX1A has been reported as tumor enhancer in brain cancers and small cell lung cancers, where its expression plays an important role in tumor formation. Increased expression of *STX1A* in neuronal cells was reported to be responsible for tumor formation in primary brain tumors (Ulloa *et al.*, 2015). However, in some other cancers, like breast cancer, the expression of *STX1A* has been shown to be variable (Fernández-Nogueira *et al.*, 2015). Our results showed the increased expression of both genes in bladder tumors compared to their normal adjacent tissue.

SNARE proteins are not only involved in the transport of neurotransmitters and neuropeptides, but also in the transfer of growth factors, recycled receptors, or integrins, and are involved in the secretion of matrix proteases that give the cell the capacity for invasion and migration. Thus, they are involved in cell progression in a regulative manner (Enrich *et al.*, 2015). Apart from cell progression, STX1A and VAMP2 have been reported to be regulatory proteins of the SNARE complex, involved in cell navigation and migration and, hence, metastasis of cancer cells (Zylbersztejn and Galli, 2011; Friedl and Alexander, 2012). The Cancer Genome Atlas (TCGA) dataset of 406 bladder tumor samples revealed an average FPKM value of 3.3 for *STX1A* expression and 14.9 for *VAMP2* expression (https://cancergenome.nih.gov). The Human Protein Atlas (HPA) and Genotype-Tissue Expression Dataset (GTEx) demonstrate a similar trend of expression for both genes in bladder cancer. Our data suggests that the expression of *STX1A* and *VAMP2* was higher in high grade tumors exhibiting aggressive behavior. Overexpression of both genes in tumor cells suggests enhanced vesicular exocytosis that might have caused increased recycling of integrins and excretion of matrix proteases, resulting in a favorable tumor microenvironment for cancer cell development and metastasis.

## Conclusions

As an important part of the core SNARE complex, STX1A and VAMP2 are associated with vesicular trafficking of growth factors, integrins, and proteases. Dysregulation of vesicular trafficking might cause multiple malignancies, more importantly cancer cell formation, altered cell adhesion, and alteration of the extracellular matrix, favoring tumor growth (Rainero *et al.*, 2013). Vesicular trafficking is supported by F-actin., and STX1A and VAMP2 were shown to interact with F-actin for SNARE-dependent exocytosis (Daniel *et al.*, 2002; Nevins and Thurmond, 2005). Enhanced expression of *STX1A* and *VAMP2*, as shown here in bladder cancer and in previous studies in other cancers (Grabowski *et al.*, 2002; Steffen *et al.*, 2008; Meng and Wang, 2015) suggest their involvement in abnormal vesicular trafficking that might have a critical role in tumor formation and metastasis.

## Supplementary material

The following online material is available for this article:

Table S1 Wilcoxon test for Synaptobrevin 2 and Syntaxin1A gene.

Table S2 Mann-Whitney test for VAMP2 and STX1A analysis in low and high-grade. tumors.

Table S3 Tukey and Kruskall-Wallis tests for VAMP2 and STX1A gene expression.

Table S4 Spearman correlations between VAMP2 with STX1A expression.
