# Supplementary material for: Increased expression levels of Syntaxin 1A and Synaptobrevin 2/Vesicle-Associated Membrane Protein-2 are associated with the progression of bladder cancer
Source: Genet Mol Biol. 2019 Jan 21;42(1):40–7. doi: 10.1590/1678-4685-GMB-2017-0339 (PMC6428126; doi:10.1590/1678-4685-GMB-2017-0339)
Supplement: Supplementary file 1 [file 1415-4757-GMB-1678-4685-GMB-2017-0339-s001.pdf]

Supplementary Material to "Increased expression levels of Syntaxin 1A and Synaptobrevin 2/Vesicle-Associated Membrane Protein-2 are associated with the progression of bladder cancer"

Table S1 - Wilcoxon test for Synaptobrevin 2 and Syntaxin1A gene expression in tumor and adjacent control samples

Gene	Sample	N	Min	Q1	Median	Q3	Max	W	Z	*p*-Value
VAMP2	Control	27	0.0014	0.1426	0.5310	1.3371	5.8934	68.0	-2.8950	**0.00379***
	Tumor	27	0.2156	0.5079	1.6722	3.3074	4.2701			
STX1A	Control	25	0.0036	0.3888	0.7429	1.5450	3.5396	4.0	-4.2513	**0.00002***
	Tumor	25	1.1893	3.6546	5.5022	9.3598	14.9647			
