# Supplementary material for: Increased expression levels of Syntaxin 1A and Synaptobrevin 2/Vesicle-Associated Membrane Protein-2 are associated with the progression of bladder cancer
Source: Genet Mol Biol. 2019 Jan 21;42(1):40–7. doi: 10.1590/1678-4685-GMB-2017-0339 (PMC6428126; doi:10.1590/1678-4685-GMB-2017-0339)
Supplement: Supplementary file 2 [file 1415-4757-GMB-1678-4685-GMB-2017-0339-s002.pdf]

Supplementary Material to "Increased expression levels of Syntaxin 1A and Synaptobrevin 2/Vesicle-Associated Membrane Protein-2 are associated with the progression of bladder cancer"

Table S2 - Mann-Whitney test for VAMP2 and STX1A analysis in low and high grade tumors.

Gene	Sample	Grade	N	Min	Q1	Median	Q3	Max	U	Z	p-Value
VAMP2	Control	LG	9	0.0017	0.0205	0.4401	0.7259	5.8934	61.0000	-1.0030	0.3159
		HG	18	0.0014	0.1561	0.6285	1.5460	2.9921			
	Tumor	LG	9	0.2156	0.2509	0.2960	0.5202	3.5573	16.0000	-3.3175	**0.0009***
		HG	18	1.1286	1.4110	2.6252	3.5141	4.2701			
STX1A	Control	LG	8	0.1698	0.4572	0.7858	1.7316	1.9603	75.0000	0.3787	0.7049
		HG	17	0.0036	0.3714	0.7159	1.5450	3.5396			
	Tumor	LG	8	1.1893	1.6221	1.9467	3.9375	4.2067	2.0000	-3.8157	**0.0001***
		HG	17	3.8966	5.1424	8.6602	10.2916	14.9647			

*At the 0.05 level, the two distributions are significantly different.
