# Supplementary material for: Increased expression levels of Syntaxin 1A and Synaptobrevin 2/Vesicle-Associated Membrane Protein-2 are associated with the progression of bladder cancer
Source: Genet Mol Biol. 2019 Jan 21;42(1):40–7. doi: 10.1590/1678-4685-GMB-2017-0339 (PMC6428126; doi:10.1590/1678-4685-GMB-2017-0339)
Supplement: Supplementary file 3 [file 1415-4757-GMB-1678-4685-GMB-2017-0339-s003.pdf]

Supplementary Material to "Increased expression levels of Syntaxin 1A and Synaptobrevin 2/Vesicle-Associated Membrane Protein-2 are associated with the progression of bladder cancer"

Table S3A - Tukey test for specific comparisons of VAMP2 and STX1A gene expression in different tumor stages

Gene	Sample	Compared stages	Mean Diff	SEM	q Value	LCL	UCL	*p*-value
VAMP2	Control	II vs I	-0.16927	0.71489	0.33486	-1.96511	1.62657	0.96961
		III vs I	0.17742	0.69475	0.36115	-1.56783	1.92266	0.96475
		III vs II	0.34669	0.69475	0.70571	-1.39855	2.09194	0.87253
	Tumor	II vs I	1.62099	0.52956	4.32894	0.29072	2.95127	**0.01518***
		III vs I	2.01919	0.51464	5.54867	0.72639	3.31198	**0.00202***
		III vs II	0.3982	0.51464	1.09423	-0.8946	1.69099	0.72268
STX1A	Control	II vs I	-0.42173	0.39081	1.52613	-1.40346	0.55999	0.53661
		III vs I	0.37488	0.3798	1.39589	-0.57919	1.32894	0.59253
		III vs II	0.79661	0.3798	2.96627	-0.15745	1.75067	0.11347
	Tumor	II vs I	4.38413	1.27859	4.84917	1.17226	7.596	**0.00651***
		III vs I	6.56977	1.24256	7.47732	3.44839	9.69114	**0.00008***
		III vs II	2.18564	1.24256	2.48756	-0.93574	5.30701	0.20659

*indicates that the difference in means is significant at the 0.05 level.

Table S3B - Kruskal-Wallis ANOVA test for the analysis of VAMP2 and STX1A gene expression in different tumor stages.

Sample	Gene	Tumor stage	N	Min	Q1	Median	Q3	Max	Chi-Square	*p*-value
Control	VAMP2	I	8	0.0017	0.0161	0.3060	0.8224	5.8934	1.3339	0.5133
		II	8	0.0014	0.0787	0.5447	1.3528	2.6478		
		III	9	0.0774	0.1567	0.6803	2.4791	2.9921		
	STX1A	I	8	0.1698	0.4572	0.7858	1.7316	1.9603	3.2751	0.1945
		II	8	0.0036	0.2035	0.4861	0.8879	1.4613		
		III	9	0.3055	0.3863	1.0950	1.9895	3.5396		
Tumor	VAMP2	I	8	0.2156	0.2477	0.3061	0.5264	3.5573	9.8077	**0.0074***
		II	8	1.3839	1.7580	2.3255	2.8434	3.5374		
		III	9	1.1286	1.2477	3.3074	3.6770	4.2701		
	STX1A	I	8	1.1893	1.6221	1.9467	3.9375	4.2067	15.6444	**0.0004***
		II	8	3.8966	4.4441	5.5794	10.1228	11.7438		
		III	9	4.2752	7.1879	9.1110	10.2916	14.9647		

*At the 0.05 level, the two distributions are significantly different.
