# Supplementary material for: Increased expression levels of Syntaxin 1A and Synaptobrevin 2/Vesicle-Associated Membrane Protein-2 are associated with the progression of bladder cancer
Source: Genet Mol Biol. 2019 Jan 21;42(1):40–7. doi: 10.1590/1678-4685-GMB-2017-0339 (PMC6428126; doi:10.1590/1678-4685-GMB-2017-0339)
Supplement: Supplementary file 4 [file 1415-4757-GMB-1678-4685-GMB-2017-0339-s004.pdf]

Supplementary Material to "Increased expression levels of Syntaxin 1A and Synaptobrevin 2/Vesicle-Associated Membrane Protein-2 are associated with the progression of bladder cancer"

Table S4 - Spearman correlations between VAMP2 with STX1A expression in tumor and adjacent control samples.

		VAMP2 Control	STX1A Control	VAMP2 Tumor	STX1A Tumor
VAMP2 Control	Spearman Corr.	1	**0.42923***	-0.06899	0.09385
	p-value	--	**0.03226**	0.73242	0.65546
STX1A Control	Spearman Corr.	**0.42923***	1	0.02538	0.02692
	p-value	**0.03226**	--	0.90413	0.89835
VAMP2 Tumor	Spearman Corr.	-0.06899	0.02538	1	**0.54769***
	p-value	0.73242	0.90413	--	**0.0046**
STX1A Tumor	Spearman Corr.	0.09385	0.02692	**0.54769***	1
	p-value	0.65546	0.89835	**0.0046**	--

two-tailed test of significance was used.

*Correlation is significant at the 0.05 level
